# Supplementary figures and images for: High prevalence of extrapulmonary tuberculosis in dairy farms: Evidence for possible gastrointestinal transmission
Source: PLoS One. 2021 Mar 30;16(3):e0249341. doi: 10.1371/journal.pone.0249341 (PMC8009431; doi:10.1371/journal.pone.0249341)

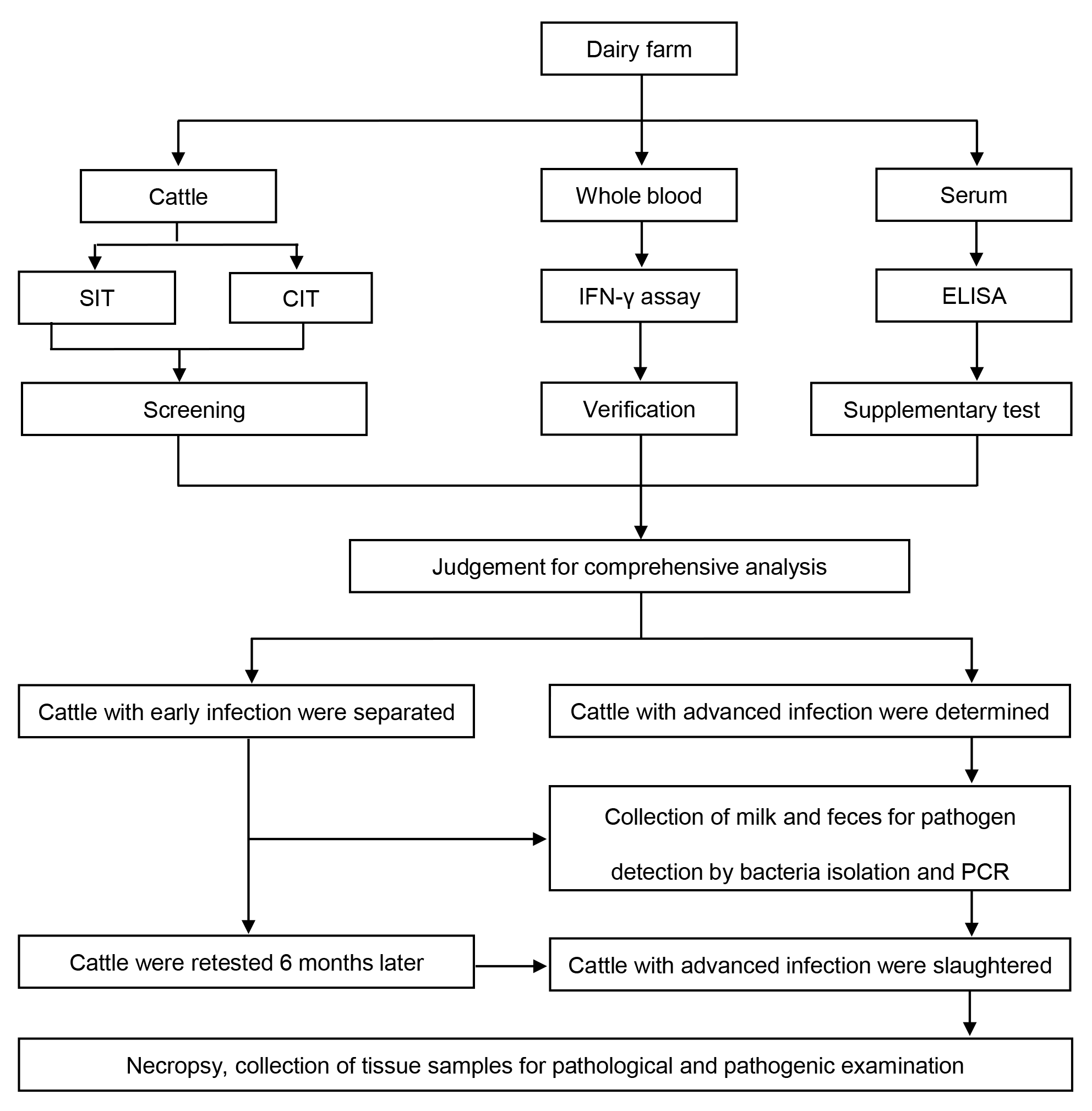

Supplement: S1 Fig — SIT, single intradermal test; CIT, comparative intradermal test; IFN-γ, gamma-interferon; ELISA, enzyme-linked immunosorbent assay; PCR, polymerase chain reaction. (TIF) [file pone.0249341.s001.tif]

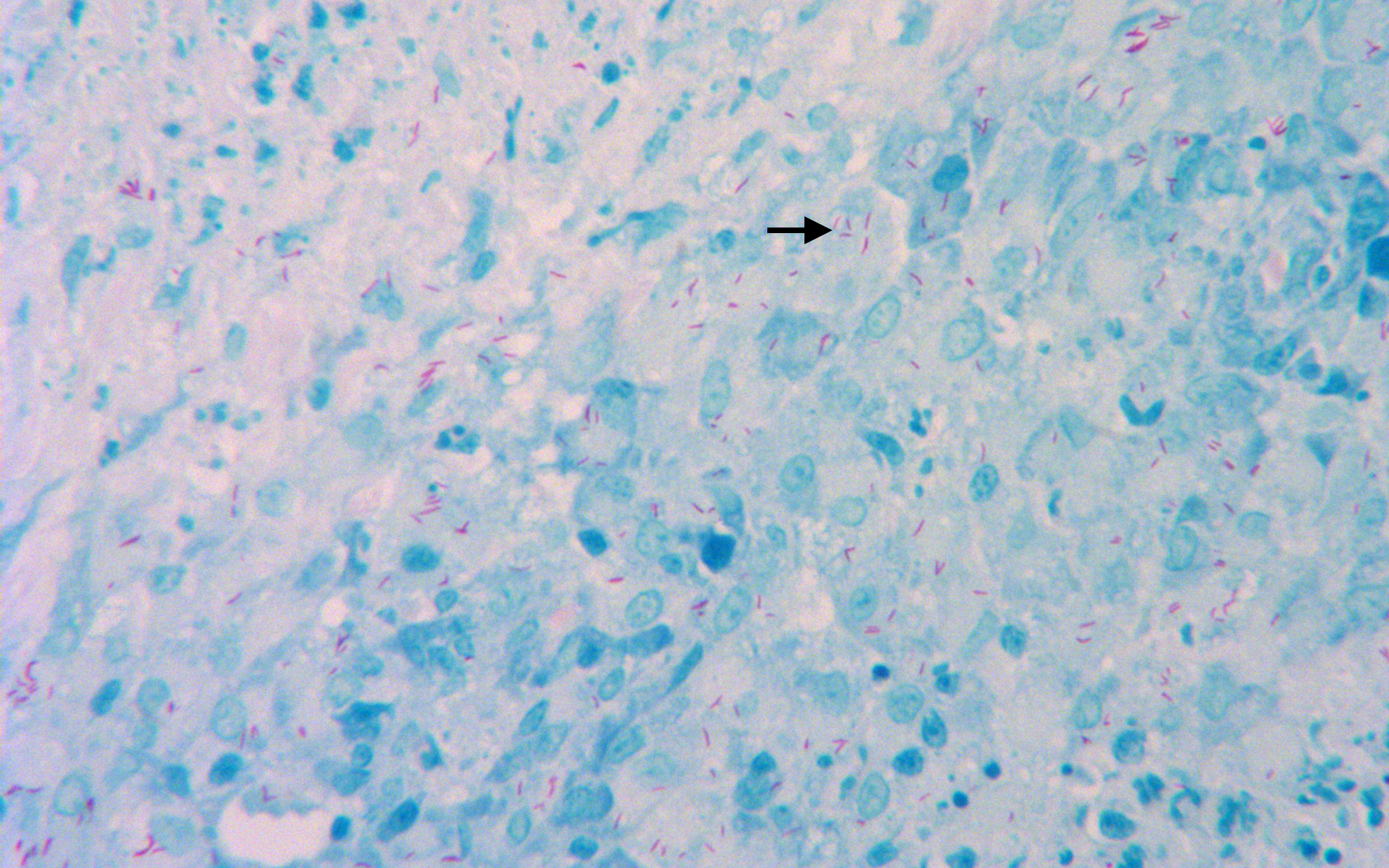

Supplement: S2 Fig — Sections were stained by Ziehl-Neelsen acid-fast staining and images were captured and shown at ×400. Acid fast bacilli were stained with red and were observed under microscope. (TIF) [file pone.0249341.s002.tif]

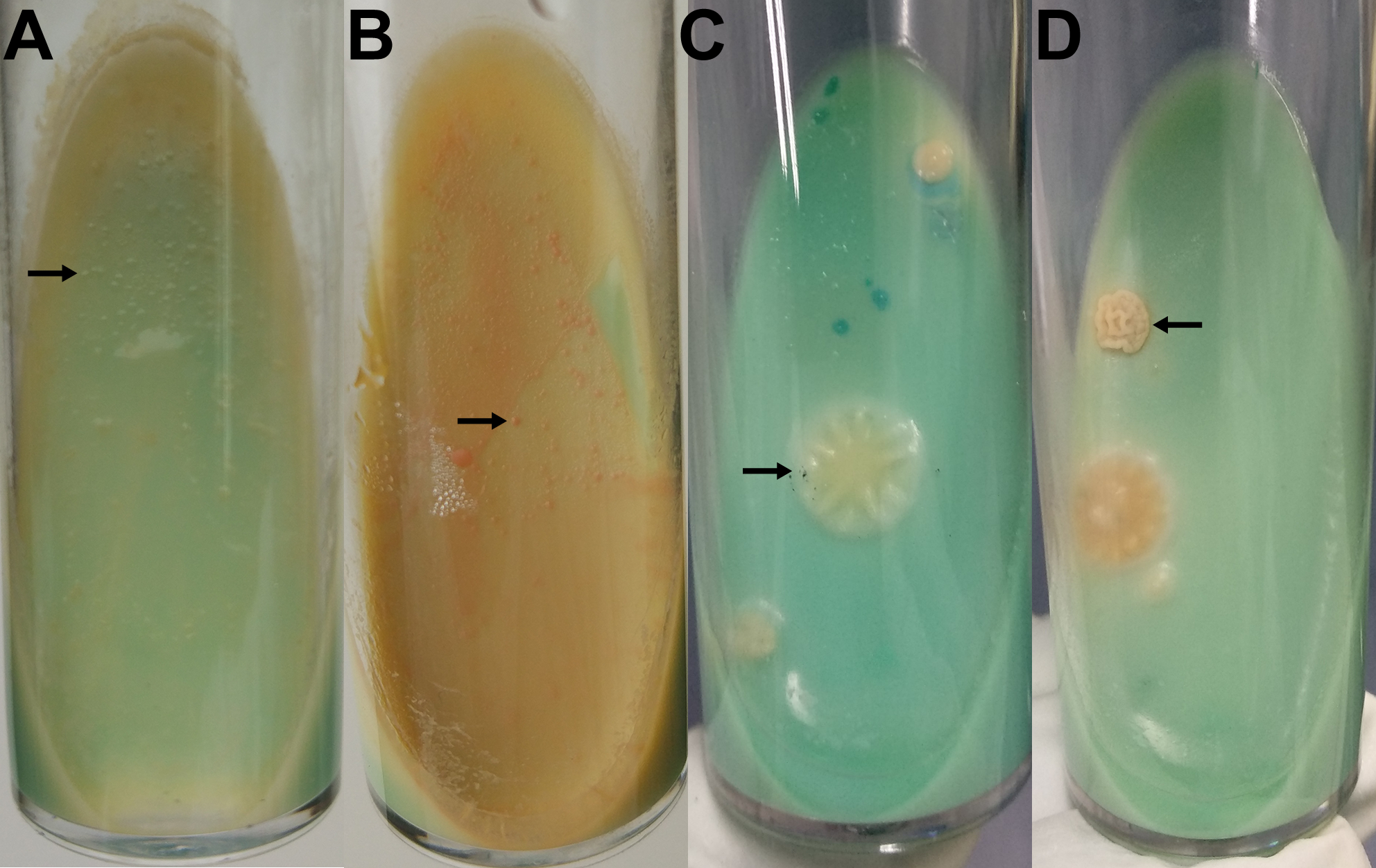

Supplement: S3 Fig — (A) Creamy-white pellet colony. (B) Beige granular colony. (C) Nodular colony. (D) Cauliflower like colony. (TIF) [file pone.0249341.s003.tif]

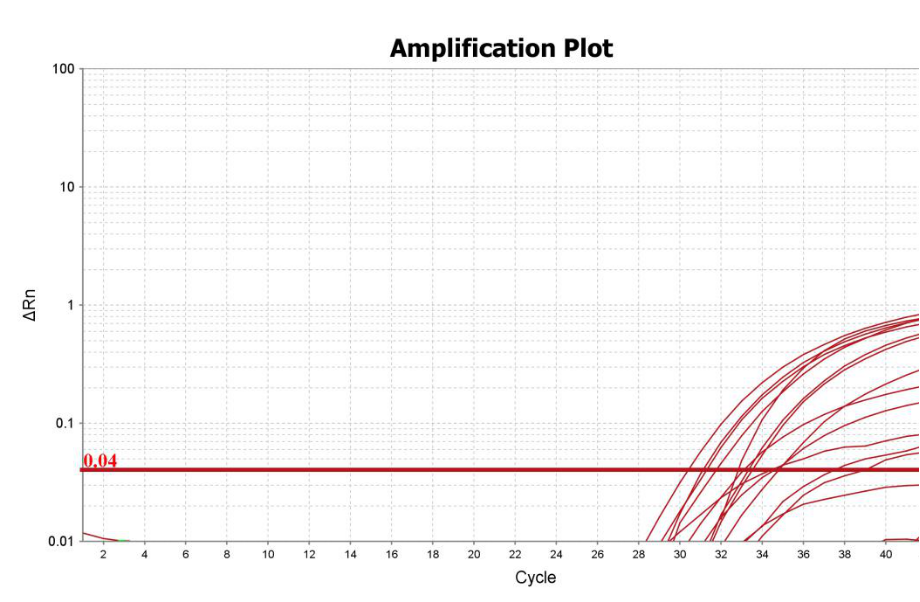

Supplement: S4 Fig — (TIF) [file pone.0249341.s004.tif]
