# Supplementary material for: High prevalence of extrapulmonary tuberculosis in dairy farms: Evidence for possible gastrointestinal transmission
Source: PLoS One. 2021 Mar 30;16(3):e0249341. doi: 10.1371/journal.pone.0249341 (PMC8009431; doi:10.1371/journal.pone.0249341)
Supplement: Attachment — Submitted filename: Response to Editor.docx [file pone.0249341.s005.docx]

Dear Dr. Fodor,

Sincerest thanks for your response and reviewers comments on our manuscript. We sincerely apologize for the great time it has taken us to respond to the issues (due to force majeure with first author). We have addressed each of the issues as outlined below. Furthermore we have added the information about the issues in “Ethical statement” section (Lines 203-205, page 9-10) of the manuscript and we hope that this comply with the referee’s remarks. For the detailed response, please see below as highlighted in blue.

**Replies to the editor:**

1) Were the skin tests and tissue collection performed as part of routine veterinary monitoring, or were they performed specifically for this study?

**Response: Thanks. Skin tests and tissue collection were included as part of routine veterinary monitoring in our national reference laboratory for bovine tuberculosis.**

2) Did you receive permission from the farm owners to test their cattle?

**Response: Thanks. The sample collecting and subsequent detection were conducted after the permission of farmer owners.**

Thank you again for your positive comments and valuable suggestions to improve the quality of our manuscript.
