# Supplementary material for: High prevalence of extrapulmonary tuberculosis in dairy farms: Evidence for possible gastrointestinal transmission
Source: PLoS One. 2021 Mar 30;16(3):e0249341. doi: 10.1371/journal.pone.0249341 (PMC8009431; doi:10.1371/journal.pone.0249341)
Supplement: Attachment — Submitted filename: Response to Reviewers.docx [file pone.0249341.s006.docx]

Dear Prof. Quinn and Reviewers,

Thanks very much for your letter and for the reviewers’ comments concerning our manuscript entitled “High prevalence of extrapulmonary tuberculosis in dairy farms: evidence for possible gastrointestinal transmission” (ID: PONE-D-20-23226R1). I really appreciate for all your comments and suggestions. We made revision as required with changes marked in red. We also made point-to-point response as highlighted in blue.

**Replies to the reviewers’ comments:**

**Reviewer #1:**

1. Did you collect the cervical lymph nodes and lung associated lymph nodes? if not, can you please explain why? I'm asking this question because very often TB disease in cattle is contained in the lymph nodes and does not progress to other organs and tissues, so it is important to examine those specific lymph nodes for gross visible lesions (in addition to the other lymph nodes you mentioned you have examined).

**Response: That’s a good point. We examined lymph nodes and numerous organs and tissues in order to identify gross visible lesions, and we then collected all the organs and tissues with lesions, including the cervical lymph nodes and lung associated lymph nodes, such as submandibular lymph nodes and hilar lymph nodes.**

2. Below few typos and suggestions.

Line 29: were tested

Line 30: the results....had advanced infection of bTB

Line 34: replace of note by in fact

Line 38-39: the phylogenetic results... (reword this sentence please)

Line 60 The Single Intradermal Test (SIT) and IFN-γ assay are the current standard diagnostic

diagnosis for bTB in some cattle farms with good economic conditions: reword

Line 69: what do you mean by harmless treatment

Line 72: is frequently overlooked

Line 82: ...and extrapulmonary bTB....

Line 126 to 129: Reword this sentence

**Response: Thank you for the suggestions. We made modification according to the previous comments. We double checked the spelling and improved the language.**
