# Supplementary material for: High prevalence of extrapulmonary tuberculosis in dairy farms: Evidence for possible gastrointestinal transmission
Source: PLoS One. 2021 Mar 30;16(3):e0249341. doi: 10.1371/journal.pone.0249341 (PMC8009431; doi:10.1371/journal.pone.0249341)
Supplement: Attachment — Submitted filename: Response to Reviewers.docx [file pone.0249341.s007.docx]

Dear Prof. Quinn and Reviewers,

Thanks very much for your letter and for the reviewers’ comments concerning our manuscript entitled “High prevalence of extrapulmonary tuberculosis in dairy farms: evidence for possible gastrointestinal transmission” (ID: PONE-D-20-23226R2). We really appreciate all your comments and suggestions. We revised our manuscript, and we have sent the revised manuscript, and a version containing all the changes to be visible. We also made point-to-point response as highlighted in blue.

**Replies to the reviewers’ comments:**

**Reviewer #1:**

1. Below few typos and suggestions.

Line 30_31: "The result indicated that 752 (5.64%) had advanced infection of bTB"

Line 39: delete "positive"

Line 41_42: "Our data demonstrate that the increase of EPTB transmitted by digestive tract is implicated in the current high prevalence rate of bTB in China"

Line 50: delete "concerne"

Line 61: "the comparative intradermal test (CIT) is also used often."

Line 65-66: "as well as interfered **with** the implementation of culling policy on the cattle suspected to be infected with bTB."

Line 132-133: I suggest to rewrite this part of the sentence as follow:

", were slaughtered and anatomical examination was performed in the local slaughterhouses named Kaerwan (Xinjiang), Musulin (Shandong), and Chengcheng (Guangxi)."

Line 139: anatomical examination was or anatomical examinations were

please modify accordingly

Line 150: "approximately"

**Response: Thank you for the suggestions. We modified and reworded the sentences according to the previous comment.**

2. Line 62 to 64: here you mentioned studies showing that PPD positive cattle don't present typical Tb associated symptoms, and they don't possess lung lesions upon necropsy. Can you please reference those studies.

**Response: Thanks. We had listed the references behind the sentence.**

3. Line 300-302: "Similar to milk samples, M. bovis nucleic acid was detected in all eight samples from fecal pool and from 34 positive cattle"

In the last sentence, which specific samples from 34 positive cattle are you talking about here? are they also fecal samples, or milk? please specify

**Response: Thank you for the suggestion. We modified the sentences according to the previous comment. In the last sentence, we had specified the 34 feces samples from bTB-positive cattle.**

4. In addition, I highly suggest detailed english editing of the manuscript, by an english native speaker or a professional editing service.

**Response: Thank you for the suggestion. We modified other spelling and improved the language. English expression has been carefully improved throughout the manuscript. And we made a marked-up copy of manuscript that highlights changes to the original version named 'Revised Manuscript with Track Changes'.**

**Special thanks to you for your good comments.**

**Thanks. We made revision.**
